# Front-of-pack nutrition labels: an equitable public health intervention

**DOI:** 10.1038/s41430-022-01205-3

**Published:** 2022-09-09

**Authors:** Simone Pettigrew, Michelle I. Jongenelis, Serge Hercberg, Chantal Julia

**Affiliations:** 1 The George Institute for Global Health, University of New South Wales, Sydney, NSW Australia; 2 Melbourne Centre for Behaviour Change, Melbourne School of Psychological Sciences, The University of Melbourne, Parkville, VIC 3010 Australia; 3 Sorbonne Paris Nord University, Nutritional Epidemiology Research Team (EREN), Epidemiology and Statistics Research Center- Université Paris Cité, Bobigny, France; 4 Public Health Department, Avicenne Hospital, AP-HP, Bobigny, France

**Keywords:** Risk factors, Education

## Abstract

It is important for nutrition interventions to be equitable to ensure they do not widen socioeconomic health-based inequalities. The role of front-of-pack nutrition labels is to provide accessible and easily understood information on product packages, and it is essential that such labels assist those who are least able to access and interpret other forms of nutrition information. This secondary analysis of the FOP-ICE food labelling study involving 18 countries (*N* = 18,393) assessed whether five different front-of-pack labels varied in effectiveness according to income status. The two outcome variables were objective understanding of products’ nutritional quality and product choice. While there were substantial differences in the ability of individual labels to improve understanding, for each label and across all labels combined there were no significant differences in changes in both outcome variables by income category. The results provide evidence that interpretive front-of-pack nutrition labels are an equitable and useful nutrition intervention.

## Introduction

Front-of-pack labels (FoPLs), especially those with interpretive designs, are recommended by the World Health Organization as a cost-effective nutrition intervention to improve diets at the population level [1]. FoPLs aim to provide consumers with accessible and easy-to-understand nutrition information to assist them make more informed food choice decisions and encourage food manufacturers to improve the nutritional quality of their products [2]. A primary advantage of FoPLs is their simplified information compared to the more detailed nutrition facts panels often located on the back of packaged foods. Higher income and education levels are associated with healthier diets [3, 4], indicating the need for simple and accessible nutrition information to ensure all consumers can understand product healthiness. However, there is evidence indicating that those of higher socioeconomic position may be better able to understand and use FoPLs [5, 6], which has the potential to exacerbate health inequities. It is therefore critical to examine the extent to which FoPLs in general and specific types of FoPLs are effective for consumers experiencing varying levels of disadvantage.

The aim of this study was to investigate whether FoPLs produce differential effects among consumers according to income level to assess whether they constitute an equitable intervention. This was done by examining the effects of FoPLs individually and in aggregate. Secondary analyses were conducted on data from 18 countries as part of the FOP-ICE project, which assessed the ability of five FoPLs (the Health Star Rating, Multiple Traffic Lights, Nutri-Score, Reference Intakes (also known as Guideline Daily Amounts), and Warning Labels) to improve (i) consumers’ understanding of the nutritional quality of food products and (ii) their food choices [7, 8]. Previous FOP-ICE data analyses have assigned income as a control variable, and as such have not attempted to analyse differences in outcomes by income category.

## Methods

The FOP-ICE data collection protocol received institutional ethics clearance and is registered at http://www.ANZCTR.org.au/ACTRN12618001221246.aspx. Briefly, an ISO-accredited web panel provider administered an online survey to ~1000 respondents from each of the following countries: Argentina, Australia, Belgium, Bulgaria, Canada, Denmark, France, Germany, Italy, Mexico, the Netherlands, Poland, Portugal, Singapore, Spain, Switzerland, the UK, and the USA (*N* = 18,393).

Quotas were used to recruit samples equivalised by gender (50% females), age (one-third in each category: 18–30, 31–50, 51+), and household income (one-third low, medium, high). Income category was calculated by estimating the median household income per country and constructing a bracket of +/−33% around the median to create the medium-income band. Values below the medium band were categorised as low income and values above as high income. Other assessed consumer characteristics included education level, whether they were a regular grocery buyer for their household, self-reported nutrition knowledge, and self-reported diet quality.

**Fig. 1:**
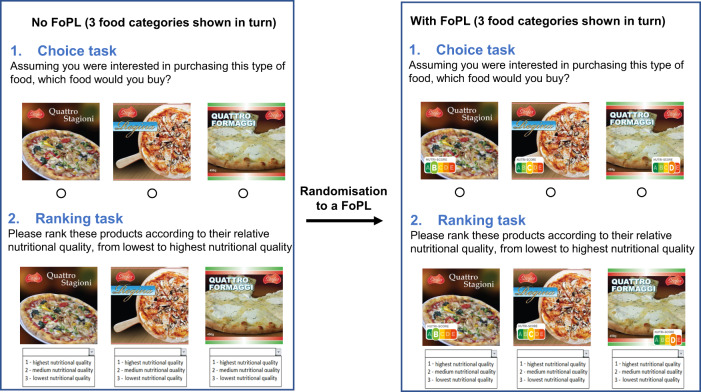
Ordering of understanding and choice tasks and front-of-pack label randomisation [7]. FoPL front-of-pack label.

The survey included an experiment measuring objective understanding (respondents’ ability to correctly rank the nutritional quality of a set of three notional products from three product categories) and product choice (using the same product choice sets as for understanding). The selected product categories (breakfast cereals, cakes, pizzas) are commonly consumed in the participating countries and have considerable nutritional variability across alternatives available in the marketplace. Figure 1 shows the ordering of the outcome variable survey items pre and post random assignment to one FoPL type. Choice was assessed prior to understanding for each product, and the ordering of product categories and individual product options was randomised to minimise priming and order effects.

Average pre- and post-exposure frequencies for correctly ranking the products by nutritional quality (yes/no) and choosing the healthiest option within the choice set (yes/no) were calculated. This dichotomous approach was taken to accommodate the ‘I don’t know’ responses that were significantly more prevalent among low-income respondents in both the pre and post FoPL exposure conditions (*p* < 0.001), with such responses considered incorrect. Two-sample *z*-tests were conducted to determine differences between income categories (low vs medium, low vs high, medium vs high) at *p* < 0.05.

## Results

Table 1 displays the proportions of respondents from each income category accurately ranking the products by nutritional quality and choosing the healthiest option pre and post FoPL exposure. Results are shown overall and by FoPL type. There were large differences in performance between FoPLs on the objective understanding outcome variable, with the Reference Intakes performing poorly compared to the interpretive FoPLs. However, there were no significant differences within FoPL category or overall by income status for both outcome variables. For all income groups, the differences in proportions correctly ranking nutritional quality pre- to post-exposure were substantially larger than the proportions making healthier product choices.

**Table 1:** Percentage pre and post exposure correctly ranking products by nutritional quality and selecting healthiest option, by income category and FoPL, averaged across the three products.

	Pre-exposure			Post-exposure			Difference		
	Low income % (*n* = 6094)	Mid Income % (*n* = 6182)	High income % (*n* = 6117)	Low income % (*n* = 6094)	Mid Income % (*n* = 6182)	High income % (*n* = 6117)	Low income % (*n* = 6094)	Mid Income % (*n* = 6182)	High income % (*n* = 6117)
All FoPLs (*N* = 18,393)									
Correct ranking	28	29	27	40	43	40	12	14	13
Selected healthiest option	36	38	39	40	43	43	4	5	4
HSR (*n* = 3680)									
Correct ranking	28	30	29	37	41	40	9	12	11
Selected healthiest option	36	37	37	40	41	43	4	4	5
MTL (*n* = 3677)									
Correct ranking	30	27	27	43	43	40	13	17	13
Selected healthiest option	35	38	38	40	45	44	5	8	5
Nutri-Score (*n* = 3678)									
Correct ranking	27	29	27	53	56	51	26	27	24
Selected healthiest option	36	37	38	41	45	44	5	7	6
Reference Intakes (*n* = 3678)									
Correct ranking	29	29	27	33	35	31	4	6	4
Selected healthiest option	36	39	39	41	43	42	5	4	3
Warning Labels (*n* = 3680)									
Correct ranking	28	28	29	37	37	38	9	9	9
Selected healthiest option	36	37	40	39	41	43	3	4	3

The three tested product categories were breakfast cereals, cakes, and pizzas.

*FoPL* front-of-pack label, *HSR* Health Star Rating, *MTL* multiple traffic lights.

## Discussion

This large international study provides evidence of the equitable nature of FoPL interventions. Across income categories, comparable effects of FoPL exposure were observed in improvements in both understanding of nutritional quality and food choices. The lack of differential effects by income category applied to all five tested FoPLs, despite their highly variable presentation formats. Consistent with previous analyses of this data set [7], and other prior work [5, 9], the pre-post exposure changes in objective understanding for respondents from all three income categories were substantially larger for the most interpretive label (the Nutri-Score) and lowest for the least interpretive label (the Reference Intakes). Changes in food choice were much smaller across all three income groups than changes in understanding [8], reflecting the imperfect relationship between knowledge and behaviour. The results highlight the need for interventions that can assist consumers use their improved nutrition knowledge to make informed and healthier choices, accompanied by comprehensive strategies that address healthy food affordability and availability [10].

The primary limitations of this study included the experimental (i.e., non-real-world) design and the inevitable priming of respondents as they moved through the survey and came to understand the experimental intent. However, these limitations applied to all respondents regardless of income level, and hence the results are useful for examining the effects of FoPL exposure for different population subgroups.

In conclusion, the findings of this study support growing evidence that interpretive FoPLs are an important component of comprehensive nutrition policies. They represent an equitable nutrition intervention, with equivalent effects found across income categories for all five assessed FoPLs.

## Data Availability

Applications for data access for non-commercial use can be made to author Chantal Julia, c.julia@eren.smbh.univ-paris13.fr.
